# The Effect of an Increase in the Rate of Multiple Births on Low-Birth-Weight and Preterm Deliveries during 1975–2008

**DOI:** 10.2188/jea.JE20100022

**Published:** 2010-11-05

**Authors:** Syuichi Ooki

**Affiliations:** Department of Health Science, Ishikawa Prefectural Nursing University, Ishikawa, Japan

**Keywords:** multiple births, low birth weight, preterm delivery, relative risk, population attributable risk percent

## Abstract

**Background:**

Despite the rapid increase in the rate of multiple births due to the growth of reproductive medicine, there have been no epidemiologic studies of the secular trends in the impact of multiple births on the rates of low-birth-weight and preterm deliveries in Japan.

**Methods:**

Japanese vital statistics for multiple live births were obtained from the Ministry of Health, Labour and Welfare and reanalyzed. With singletons as the reference group, an analysis was performed of secular trends in relative risk and population attributable risk percent of low-birth-weight (<2500 grams), very-low-birth-weight (<1500 grams), and extremely-low-birth-weight (<1000 grams) deliveries, using 1975–2008 vital statistics, and of preterm deliveries (ie, before 37, 32, and 28 weeks), using 1979–2008 vital statistics.

**Results:**

The rate of multiple births doubled during the past 2 decades, and about 2% of all neonates are now multiples. The population attributable risk percent tended to increase during the same period for all variables, and was approximately 20% in 2008.

**Conclusions:**

The public health impact of the rapid increase in multiple births remains high in Japan.

## INTRODUCTION

Numerous studies have shown that the increasing use of assisted reproductive technologies (ART) and rising maternal age have resulted in an increase in multiple births in all developed counties.^1^^–^^10^ Increasing twinning rates in Japan have also been attributed to the higher proportion of mothers treated with ovulation-inducing hormones and in vitro fertilization.^11^

Multiple births are associated with a high risk of preterm birth and low birth weight.^12^^–^^15^ Preterm newborns account for a high percentage of perinatal mortality^16^ and are at increased risk for health and developmental problems if they survive. Studies on the effects of multiple births have revealed a significant influence on pregnancy and long-term outcomes. Higher prevalences of cerebral palsy,^17^^,^^18^ sudden infant death syndrome,^19^ attention deficit hyperactive disorder,^20^ and other disorders^21^^,^^22^ were reported. Delays in physical growth^23^^,^^24^ and motor^25^^,^^26^ and language^27^^,^^28^ development as compared with singletons have also been frequently reported. Child abuse is also reported to be more frequent in families with multiples.^29^ Furthermore, preterm and low-birth-weight infants are more likely to require costly intensive care.^30^^–^^33^

Studies have examined the impact of multiple births on birth weight and preterm delivery.^3^^,^^10^ However, because these studies were conducted in Western countries, it is useful to examine the current situation with respect to multiple births in Japan, since policies and guidelines on fertility treatment, especially concerning multiple births, considerably differ from those of Western countries. For example, Scandinavian countries and Belgium adopted an elective single embryo transfer (SET) policy for ART.^34^^,^^35^ In these countries, even twin births have been decreasing in the past few years. In 2008, the Japanese Society of Obstetrics and Gynecology established a SET policy to avoid multiple births. The availability of health insurance for fertility treatment also affects the rates of multiple births.^36^ Health insurance in Japan does not usually cover fertility treatment.

The aim of this study was to analyze secular trends in the impact of multiple births on low-birth-weight and preterm deliveries in Japan.

## METHODS

All available vital statistics on multiple births in the entire Japanese population since 1975—assembled by the Ministry of Health, Labour and Welfare—were collected and reanalyzed. The vital statistics are a complete survey based on birth records and are published as an annual report of aggregate, not individual, data. The number of all registered live births with respect to plurality (1 or more) and birth weight (<2500 grams, <1500 grams, and <1000 grams) were collected for 1975–2008; data on gestational weeks (before 37, 32, and 28 weeks) were collected for 1979–2008. As infants in multiple births were not differentiated with respect to birth weight or gestational weeks, these babies were grouped into 1 category, as multiples. No lower gestational age or birth weight criteria were applied, so as to exclude extremely preterm and very small newborns.

First, secular trends in the rate of multiple births were assessed to determine the current situation in Japan. The rate of multiple births was defined as the proportion of live multiple births among all live births, including multiples. In this calculation, multiple births were thus treated as individual neonates. In other words, if a pair of twins were both born alive, the pair was counted as 2 neonates.

Next, secular trends in the relative risk (RR) and population attributable risk among multiple births of low birth weight (LBW: <2500 grams), very low birth weight (VLBW: <1500 grams), and extremely low birth weight (ELBW: <1000 grams) were assessed, with singletons as the reference group, using 1975–2008 vital statistics; preterm deliveries (before 37, 32, and 28 weeks) were assessed using 1979–2008 vital statistics. RR and PAR% were defined using the following formulas,^37^ where P denotes the prevalence of multiple births, ie, the rate of multiple births:

$$\begin{array}{l} \text{RR=prevalence of low-birth-weight or preterm}\\ \text{deliveries in multiples/prevalence of}\\ \text{low-birth-weight or preterm deliveries in singletons} \end{array}$$

PAR%=P×(RR-1)/{P×(RR-1)+1}×100

The prevalence (proportion) was used instead of incidence for all calculations. Although RR is by definition a comparison of the incidence in exposed and unexposed groups, it is very difficult to use this parameter for birth data. The limitations of this definition will be discussed later.

## RESULTS

**Figure 1.**
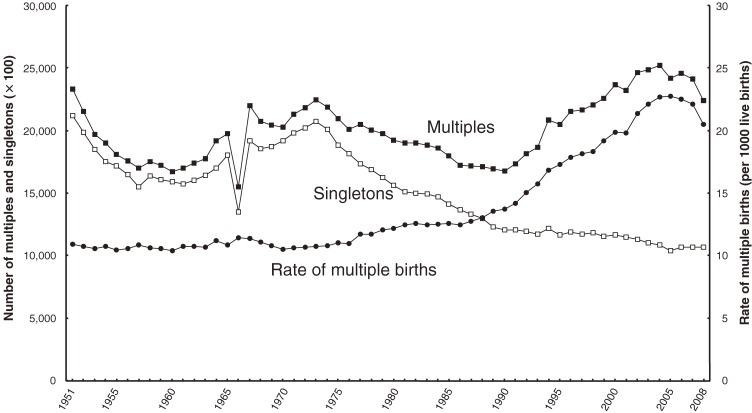
Secular trends in the numbers of liveborn singletons and multiples and the rate of multiple births

Secular trends in the rate of multiple births from 1951 through 2008 are shown in Figure 1. The rate of multiple births began to markedly increase in the mid-1980s, although it slightly decreased in the last 3 years of the data.

**Figure 2.**
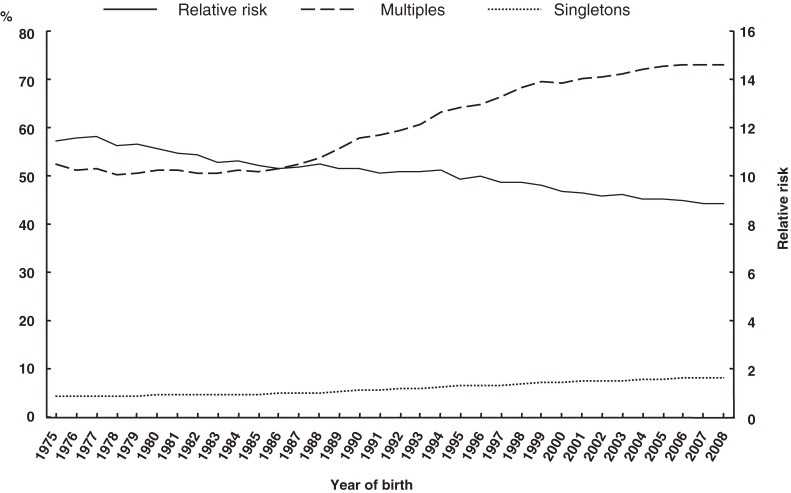
Secular trends in low birth weight in multiples and singletons, and in relative risk

**Figure 3.**
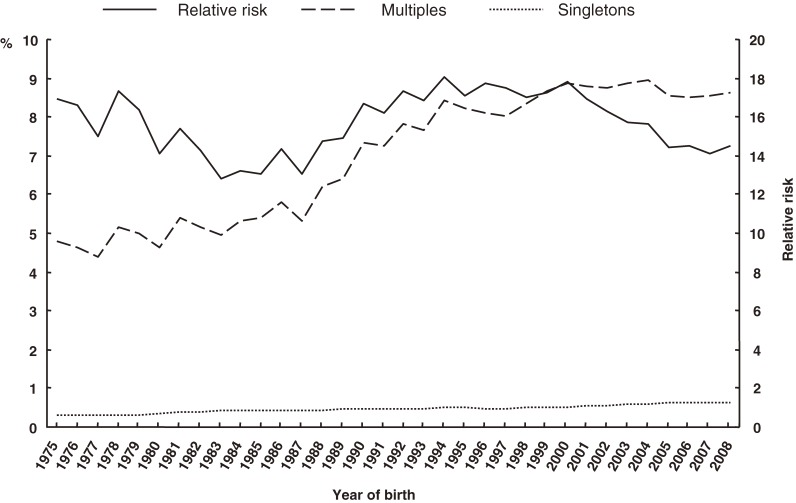
Secular trends in very low birth weight in multiples and singletons, and in relative risk

**Figure 4.**
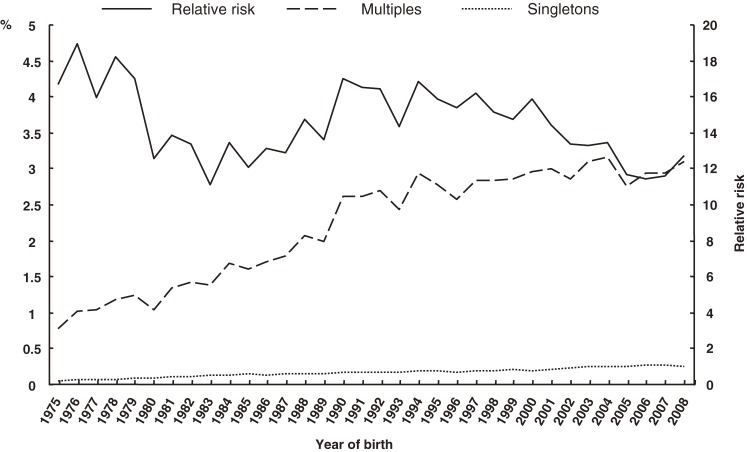
Secular trends in extremely low birth weight in multiples and singletons, and in relative risk

The percentages of singleton and multiples in the 3 low-birth-weight classifications and RRs are shown in Figures 2–4. The proportions of multiples with LBW, VLBW, and ELBW tended to increase over time; however, the RRs did not.

**Figure 5.**
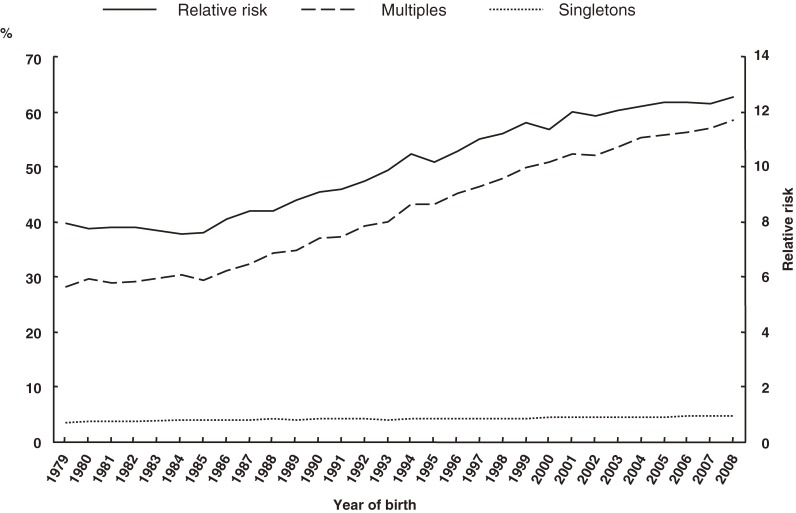
Secular trends in preterm delivery before 37 weeks in multiples and singletons, and in relative risk

**Figure 6.**
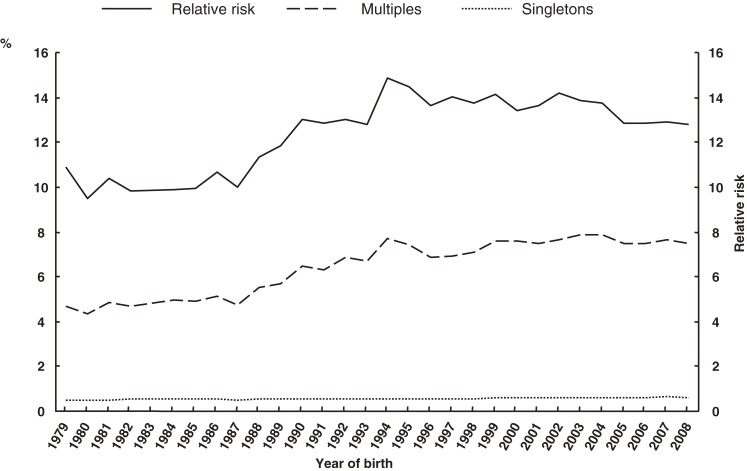
Secular trends of preterm delivery before 32 weeks in multiples and singletons, and in relative risk

**Figure 7.**
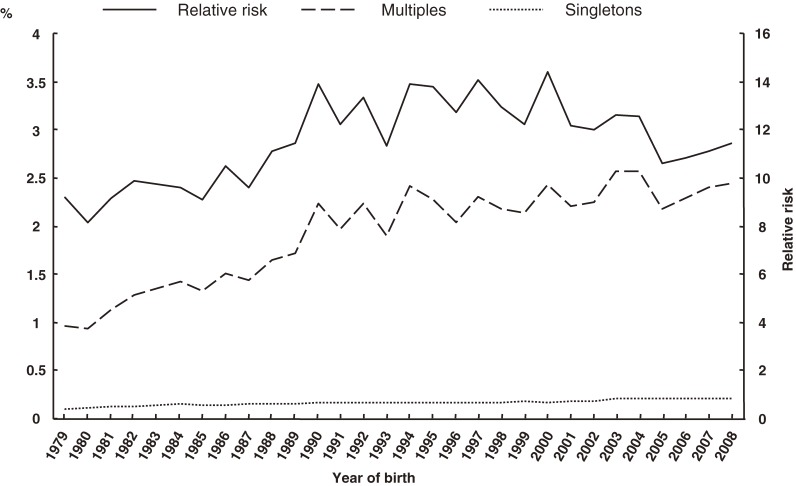
Secular trends in preterm delivery before 28 weeks in multiples and singletons, and in relative risk

The percentages of preterm deliveries before 37, 32, and 28 weeks, with respect to plurality, and RRs are shown in Figures 5–7, respectively. The proportion of preterm deliveries before 37 weeks in multiples has continuously increased. The proportions of preterm deliveries before 32 and 28 weeks have tended to increase overall. The RRs for preterm deliveries before 37, 32, and 28 weeks were all approximately 12 in 2008.

**Figure 8.**
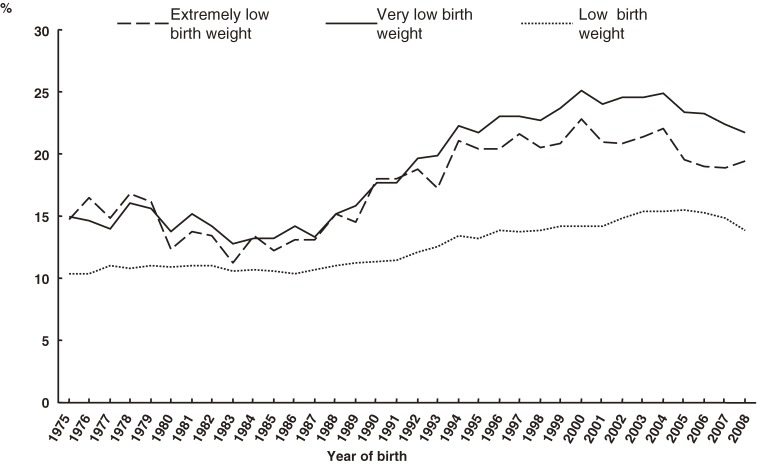
Secular trends in population attributable risk% of 3 low-birth-weight categories among multiple births

**Figure 9.**
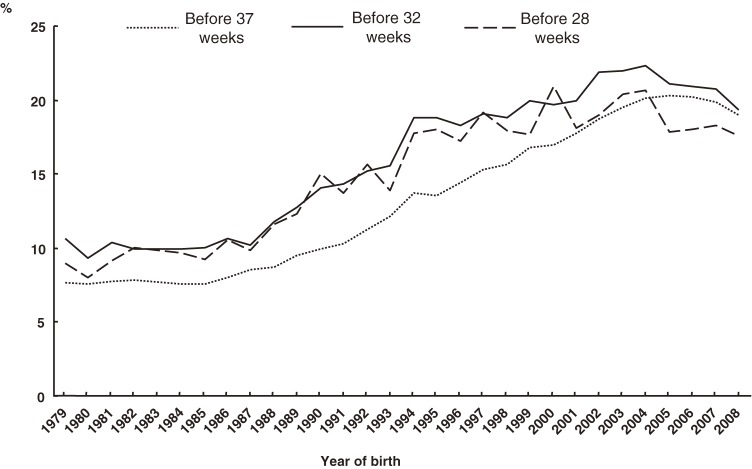
Secular trends in population attributable risk% of preterm delivery before 37, 32, and 28 weeks among multiple births

The PAR% of low-birth-weight and preterm deliveries are shown in Figures 8 and 9, respectively. PAR% tended to increase during the past 30 years in all categories. Regarding birth weight, the largest PAR% was observed in VLBW infants, followed by ELBW and LBW infants.

The PAR% of preterm delivery before 37 weeks in multiples tended to increase linearly for an extended period of time, although it has recently decreased slightly to approximately 20%. The PAR% of preterm delivery before 32 and 28 weeks were always higher than that of preterm delivery before 37 weeks, although in recent years the difference has been small.

The increase in PAR% between 1979 and 2008 is expressed as the percentage difference between the value of 2008 and that of 1979, divided by the value of 1979. The PAR% increases for birth weights under 2500 grams, 1500 grams, and 1000 grams were 26% (= (13.9 − 11.0)/11.0), 39% (= (21.7 − 15.6)/15.6), and 20% (= (19.4 − 16.1)/16.1), respectively. The PAR% increases for preterm deliveries before 37, 32, and 28 weeks were 147% (= (19.0 − 7.7)/7.7), 83% (= (19.4 − 10.6)/10.6), and 96% (= (17.6 − 9.0)/9.0), respectively.

## DISCUSSION

Although the rate of multiple births is very low in Japan,^11^^,^^38^ it approximately doubled from 1975 to 2008, which is close to the rate of increase in many Western countries.

Rates of multiple births have been decreasing in some Western countries due to medical intervention to reverse the rapid increase in iatrogenic multiple births.^34^^,^^35^ There was also a decrease in the rate of multiple births in Japan from 2006 through 2008, suggesting that the SET policy has had an effect on ART. Nevertheless, the recent decrease is slight and of short duration and thus the effects of multiple births on perinatal maternal and child health indicators remain an important public health concern.

It can be problematic to use singleton cut-off points—ie, 2500 grams and 37 weeks—to estimate intrauterine growth in multiples. The present analyses were performed using this cut-off point, however, as this has been standard practice in many previous reports on multiple births. In addition, the availability of categorical data made this the ideal cut-off point.

PAR% is a useful indicator for clarifying the public health impact of certain risk factors. This indicator is influenced not only by RR, but also by the prevalence of a risk factor, namely the rate of multiple births. Secular trends in PAR% thus reflect secular trends in both RR and the rate of multiple births.

In an international study, the PAR% of liveborn twins delivered before 37 weeks was reported to range from 10.3% (United States) to 18.7% (France), and from 13.7% (United States) to 21.3% (France) for deliveries before 33 weeks, in 1995–1997.^3^ This is in relatively good accordance with the present results for this period (13.6% to 15.3% and 18.8% to 19.1% respectively), although the present data on preterm delivery were for deliveries before 37 and 32 weeks.

Although the present data include all multiple births, about 98% were twins (data not shown). Because twins have a major population-based impact on trends in perinatal health indicators,^3^ the present results can reasonably be compared. Another international study^10^ showed that the PAR% of preterm delivery before 37 weeks among liveborn multiples ranged from 17.6% (Italy) to 24.8% (Denmark) in 1998–2001. The figures for this period were slightly lower in the present study, at 15.7% to 17.0%, which might be partly due to the lower prevalence of multiples in Japan as compared with Western countries.

In the international study mentioned above,^3^ the PAR% of twins under 2500 grams and 1500 grams was reported to be 16.6% (United States) to 21.4% (France) and 16.8% (United States) to 25.7% (France), respectively, in 1995–1997. This is in relatively good accordance with the present results (13.2% to 13.8% and 21.7% to 23.0%, respectively) for this periods, although the PAR% of LBW was slightly lower in Japan, again partly reflecting the lower prevalence of multiples in Japan.

LBW is now increasing in Japan, irrespective of plurality. The reasons are complex and include well established risk factors for low birth weight by gestational age, such as low pre-pregnancy body mass index, strict restriction of weight gain during pregnancy, and maternal smoking.^39^ However, these factors do not seem to increase the RR for LBW, using the definition of RR employed in the present study.

In addition, because the PAR% for a certain year is a mathematical function of the RR in that year, the secular trend with the largest PAR% was for VLBW, followed by ELBW and LBW, which reflects the secular trend in the RRs for VLBW, ELBW, and LBW, in that order. Although the sociobiological reasons why the PAR% of VLBW has been higher than that of ELBW during the past 15 years are unclear, it could be argued that the proportion of multiples with birth weights from 1000 grams to 1500 grams has increased during this 15-year period. Very preterm delivery and low-birth-weight newborns require intensive care in neonatal units, and are at high risk for neonatal morbidity and developmental problems.^16^^–^^28^ Therefore, the rising number of multiples will increase the burden on neonatal services and health services in general, and will result in higher numbers of children surviving with impairment.

The impact of fertility treatment on multiple births was first discussed years ago.^1^ The rapid increase of iatrogenic multiple births is now a public health concern, one that goes beyond the purely obstetric problems that occur with multiple births and the post-birth support required for some families with multiples. Nevertheless, a societal discussion that includes families with multiples, obstetric associations for fertility treatment and perinatal management, governmental offices, policy makers, and public health researchers has not occurred, at least in Japan.

The present study was performed using vital statistics because these data are extensively monitored. The results offer clear evidence of the public health impact of the rapid increase in multiple births. Other adverse outcomes related to multiple births, such as cerebral palsy,^17^^,^^18^ are also useful indicators, and should be monitored.^18^ Moreover, the societal impact of a rapid increase in multiples can be assessed from different perspective, including that of medical economics,^30^^–^^33^ laws and guidelines on fertility treatment and multiple births,^34^^,^^35^ information obtained through questionnaires or interview surveys on the child-rearing difficulties families with multiples face,^40^^–^^44^ and social family support systems or maternal and child health policies.^45^

The present study has several limitations, the most important of which is that the author could not control for confounding factors that affect birth weight and/or gestational age, such as maternal age, parity, and sex of the neonates. Another limitation is that RRs were estimated using prevalence (proportion), which establishes an upper limit for RRs. For example, even if the prevalence of LBW in multiples is 100%, the maximum RR is 10, if we assume that the prevalence of LBW among singletons is 10% (RR = 100/10). Because of this restriction, the PAR% may be underestimated, as PAR% is by definition a function of RR and P (rate of multiple births, which is constant in a certain year) and decreases with a decline in RR.

These results should prove useful for other Asian countries, where the problem of iatrogenic multiples is ongoing.^46^ Public health initiatives to resolve the many problems related to the rapid increase of multiple births are expected to be proposed or implemented.
